# The effect of antiviral therapy on serum lipid profiles in chronic hepatitis C

**DOI:** 10.18632/oncotarget.25092

**Published:** 2018-04-20

**Authors:** Batbold Batsaikhan, Ching-I Huang, Ming-Lun Yeh, Chung-Feng Huang, Nei-Jen Hou, Zu-Yau Lin, Shinn-Cherng Chen, Jee-Fu Huang, Ming-Lung Yu, Wan-Long Chuang, Jin-Ching Lee, Chia-Yen Dai

**Affiliations:** ^1^ Graduate Institute of Medicine, College of Medicine, Kaohsiung Medical University, Kaohsiung, Taiwan; ^2^ Department of Internal Medicine, Institute of Medical Sciences, Mongolian National University of Medical Sciences, Ulaanbaatar, Mongolia; ^3^ Department of Internal Medicine, Kaohsiung Medical University Hospital, Kaohsiung Medical University, Kaohsiung, Taiwan; ^4^ Faculty of Internal Medicine, College of Medicine, Kaohsiung Medical University, Kaohsiung, Taiwan; ^5^ Department of Biotechnology, College of Life Science, Kaohsiung Medical University, Kaohsiung, Taiwan; ^6^ Health Management Center, Kaohsiung Medical University Hospital, Kaohsiung Medical University, Kaohsiung, Taiwan

**Keywords:** HCV, lipid profiles, triglycerides, FIB4, antiviral treatment

## Abstract

**Background:**

Low lipid profile is associated with hepatitis C virus (HCV) infection. Chronic HCV infection is the main cause of liver injury and serum lipid levels during antiviral treatment. We aimed to evaluate the effect of antiviral treatment on the change of lipid profiles during HCV treatment.

**Methods:**

Total 863 patients who complete the interferon-based therapy in Kaohsiung Medical University Hospital were included in this study. The lipid profile measured and evaluated in baseline and after 6 months of the treatment.

**Results:**

Sustained virological response (SVR) was achieved in 81.2% of all patients. The baseline triglycerides (TG) levels in the SVR group and non SVR groups were similar. The TG levels at 6 months after cessation of the treatment was significantly elevated in SVR group (102.9±57.0 mg/dL, p=0.0001) but did not elevated in non SVR group (94.5±45.6 mg/dL, p=0.690) compared with baseline TG levels. After adjusting patients by four indexes for fibrosis (FIB4) in cut-off point 3.25, serum TG levels significantly increased in low FIB4 group (103.2±57.9 mg/dL, p=0.0001) but not in high FIB4 group (98.1±49.6 mg/dL, p=0.095) after 6 months end of the treatment. Serum TG level was increased greater in patients who had low FIB4 score and patients who achieved SVR (baseline 89.1±34.8 mg/dL; 6 months after treatment 104.3±59.3 mg/dL, paired T test p=0.0001).

**Conclusion:**

The clearance of the HCV RNA is the main determinant of the increase of lipids after PegIFN/RBV treatment. However advanced fibrosis also has an effect in increase of lipids after the treatment.

## INTRODUCTION

Liver plays fundamental role in lipid metabolism and hepatitis C virus (HCV) is linked to the lower lipid profiles and the progression of the chronic liver disease [1]. Globally over 177.5 millions of people chronically infected with HCV as a prevalence of 2.5% of World population and it is serious burden of global health [2] and the prevalence of serum anti HCV is higher in Taiwanese population [3, 4]. Several studies reported that HCV associated with lower lipid profiles and predisposes to dyslipidemia, liver steatosis or advanced fibrosis [1, 5]. Lipids also play an important role in HCV life cycle or its structure [6]. However hypobetalipoproteinemia caused by HCV binding to lipoprotein was already reported [7] and it is may be one of the main pathway to lowering lipid profiles during HCV infection. Several studies have reported dysregulated serum lipid levels in HCV infection, especially low levels of low-density lipoprotein cholesterol (LDLC) [8] and little is known about the serum triglyceride (TG) levels in HCV infection. Another study reported that TG may contribute to liver fibrosis due to deposition of TG and liver steatosis [9]. There is limited number of studies determined lipid profile influence of fibrosis stage chronic HCV infection [10]. Although protease inhibitors and other direct acting anti-viral drugs have been used in the Western countries, but the Pegylated interferon-alpha (PegIFN) and ribavirin (RBV) combination therapy for chronic hepatitis C (CHC) is still remaining the major treatment for HCV infection in many countries. We have reported a higher cure rate for PegIFN/RBV therapy in Taiwan than Western countries [11, 12]. Since the change of the lipid profiles are interesting after curing of the HCV infection and not be well-documented, we aimed to evaluate the effect of antiviral therapy on lipid profiles and to investigate the factors related to the changes of lipid profiles in CHC patients.

## RESULTS

### Factors associated with the sustained viral response

The basic characteristics and associated factors to the SVR of 863 patients are shown in Table 1. The mean age was 53.9±11.0 years and 701 (81.2%) patients achieved SVR. The factors associated with SVR were lower proportion of HCV genotype 1b (36.5%), lower HCV RNA level (1162.5±2152.2 KIU/mL), lower body mass index (24.6±3.6 kg/m^2^), lower FIB4 score (2.9±2.1) and higher platelet (173.1±61.3 10^9^/L) level. There was no significant difference in lipid profiles (Table 1). We performed multivariate logistic regression analysis and HCV genotype 1 (Odds Ratio [OR] – 0.301, 95% Confidence Interval [CI 95%] – 0.207-0.436, p=0.0001), HCV RNA viral load (OR – 1.000, CI 95% - 1.000-1.000, p=0.0001), BMI (OR – 0.920, CI 95% - 0.877-0.965, p=0.001) and FIB4 (OR – 0.837, CI 95% - 0.776-0.903, p=0.0001) were associated with antiviral treatment response (Data not shown).

**Table 1:** The comparison of basic characteristics and lipid profiles in SVR or non SVR group

Characteristics	Total	SVR	Non SVR	*P* value
**Number of patients (%)**	863	701(81.2%)	162(18.8%)	
**Age (mean, SD)**	53.9±11.0	53.3±10.9	56.5±11.2	**0.001**
**Sex** ** male** ** female**	442(51.2%) 421(48.8%)	369(52.6%) 332(47.4%)	73(45.1%) 89(54.9%)	0.082^*^
**HCV genotype (1b/others, %)**	358(41.5%)/505(58.5%)	256(36.5%)/445(63.5%)	102(63%)/60(37%)	0.0001^*^
**HCV RNA (KIU/mL, SD)**	1296.9±2242.2	1162.5±2152.2	1873.8±2520.1	**0.001**
**BMI**	24.8±3.7	24.6±3.6	25.8±3.5	**0.0001**
**Diabetes** ** Yes** ** No**	137(16%) 719(84%)	104(15%) 591(85%)	33(20.5%) 128(79.5%)	0.084^*^
**Interleukin 28 beta** ** TT** ** Other**	536(62.1%) 327(37.9%)	445(63.5%) 256(36.5%)	91(56.2%) 71(43.8%)	0.084^*^
**FIB4 (mean, SD)**	3.1±2.4	2.9±2.1	3.8±3.4	**0.001**
**GOT (U/l)**	94.0±58.1	94.7±59.9	91.0±49.6	0.458
**GPT (U/l)**	137.5±92.7	140.1±93.9	126.4±86.8	0.092
**GGT (U/l)**	60.6±58.6	60.8±60.6	59.9±49.4	0.872
**PLT (10^9^/l)**	169.8±62.4	173.1±61.3	155.9±65.3	**0.002**
**AFP (ng/ml)**	17.4±119.0	18.1±131.8	14.4±17.3	0.743
**Triglycerides (mg/dl)**	91.1±34.9	90.5±34.3	93.5±37.3	0.336
**Cholesterol (mg/dl)**	166.8±31.1	166.0±30.3	170.2±34.0	0.127
**HDLC (mg/dl)**	45.7±14.4	45.4±14.2	46.7±15.1	0.305
**LDLC (mg/dl)**	97.9±27.9	97.7±27.4	98.6±29.6	0.731

^*^P value of Chi squared test, SVR – sustained virological response, BMI – body mass index, GOT – glutamic-oxaloacetic transaminase, GPT – glutamic-pyruvic transaminase, GGT – gamma-glutamyl transferase, PLT – platelet, AFP – alpha-fetoprotein, HDLC – high density lipoprotein, LDLC – low density lipoprotein, SD – standard deviation.

### The dynamic of the serum lipid levels in SVR and non SVR patients

**Figure 1:**
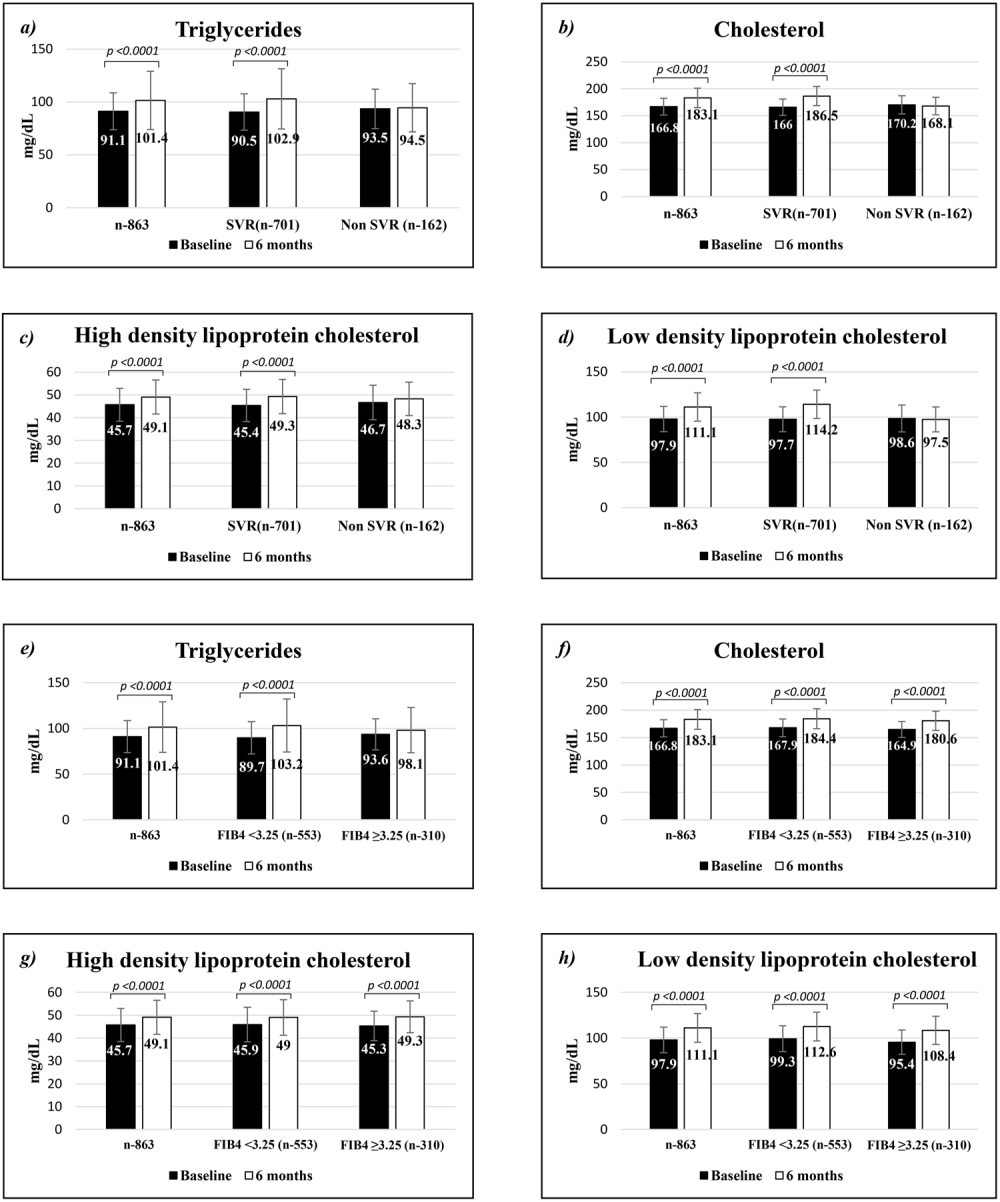
The change of serum lipid levels in baseline and after 6 months of antiviral treatment Comparison of serum **(a)** triglycerides, **(b)** cholesterol, **(c)** high density lipoprotein cholesterol and **(d)** low density lipoprotein cholesterol levels in baseline and after 6 months of antiviral treatment stratified by sustained virologic response. Comparison of serum **(e)** triglycerides, **(f)** cholesterol, **(g)** high density lipoprotein cholesterol and **(h)** low density lipoprotein cholesterol levels in baseline and after 6 months of antiviral treatment stratified by FIB4 (cut-off point 3.25).

The mean lipid levels of before and after treatment for all patients as well as for patients stratified by SVR and non SVR groups were shown in Table 2. All serum lipid levels have been significantly increased in all patients and SVR groups but not in non SVR group. However the serum lipid levels of SVR group were higher than non SVR group even it was not significant before the treatment. Patients who achieved SVR experienced a mean increase of 12.3±51.6 mg/dl of TG level, but non SVR group experienced mean increase of 1.2±43.0 mg/dl (p=0.011) (Supplementary Table 1). The similar significant difference was seen in HDLC (p=0.018), TC (p=0.0001) and LDLC (p=0.0001) levels compared to non-SVR group. Therefore serum lipid levels increased in patients who achieved SVR which is successful eradication of HCV (Figure 1a, 1b, 1c and 1d).

**Table 2:** The comparison of basic characteristics and lipid profiles in low or high FIB4 group

Characteristics	Total	FIB4 <3.25	FIB4 ≥3.25	*P* value
**Number of patients (%)**	863	553(64.1%)	310(35.9%)	
**Age (mean, SD)**	53.9±11.0	49.9±10.7	61.0±7.4	**0.0001**
**Sex** ** male** ** female**	442(51.2%) 421(48.8%)	322(58.2%) 231(41.8%)	120(38.7%) 190(61.3%)	0.0001^*^
**HCV genotype (1b/others, %)**	358(41.5%)/505(58.5%)	233(42.1%)/320(57.9%)	125(40.3%)/185(59.7%)	0.604^*^
**HCV RNA (KIU/mL, SD)**	1296.9±2242.2	1509.2±2590.3	916.2±1340.0	**0.0001**
**Viral response** **SVR** **Non SVR**	701(81.2%) 162(18.8%)	466(84.3%) 87(15.7%)	235(75.8%) 75(24.2%)	0.002^*^
**BMI (kg/m^2^)**	24.8±3.7	24.8±3.5	25.0±3.9	0.425
**Diabetes** ** Yes** ** No**	137(16%) 719(84%)	69(12.6%) 479(87.4%)	68(22.1%) 240(77.9%)	0.0001^*^
**Interleukin 28 beta** ** TT** ** Other**	536(62.1%) 327(37.9%)	356(64.4%) 197(35.6%)	180(58.1%) 130(41.9%)	0.067^*^
**GOT (U/l)**	94.0±58.1	73.4±40.1	130.9±66.5	**0.0001**
**GPT (U/l)**	137.5±92.7	124.3±85.8	161.1±99.8	**0.0001**
**GGT (U/l)**	60.6±58.6	54.6±55.2	71.5±63.0	**0.0001**
**PLT (10^9^/l)**	169.8±62.4	197.0±58.1	121.4±34.1	**0.0001**
**AFP (ng/ml)**	17.4±119.0	9.3±25.3	31.0±191.3	**0.015**
**Triglycerides (mg/dl)**	91.1±34.9	89.7±35.4	93.6±33.9	0.111
**Cholesterol (mg/dl)**	166.8±31.1	167.9±32.1	164.9±29.0	0.179
**HDLC (mg/dl)**	45.7±14.4	45.9±15.1	45.3±12.9	0.510
**LDLC (mg/dl)**	97.9±27.9	99.3±28.4	95.4±26.6	**0.047**

^*^P value of Chi squared test, SVR – sustained virological response, BMI – body mass index, GOT – glutamic-oxaloacetic transaminase, GPT – glutamic-pyruvic transaminase, GGT – gamma-glutamyl transferase, PLT – platelet, AFP – alpha-fetoprotein, HDLC – high density lipoprotein, LDLC – low density lipoprotein, SD – standard deviation.

### Factors associated with the advanced fibrosis and the dynamic of the serum lipid levels in mild or advanced fibrosis

We analyzed before and after treatment lipid levels in FIB4 stratified groups by using 3.25 as a cut-off point. The univariate analysis showed that patients with high FIB4 (over 3.25) were older; higher proportion of female patients; lower HCV RNA; high number of diabetic patients; lower SVR rate; higher GOT, GPT, GGT, AFP levels and lower platelet count (Table 2). All serum lipid levels have been significantly increased in all patients according to low and high FIB4 groups. But serum TG level in high FIB4 group was not revealed significant increase (Figure 1e, 1f, 1g and 1h). The difference of TG level before and after treatment was sensitive to in FIB4 stratified groups. The change of TG level in low FIB4 group was higher (13.5±51.9) compared to high FIB4 group (4.4±47.1; p=0.011). However the difference of TC, HDLC and LDLC levels were similar in low and high FIB4 groups. Serum TG level has an association with FIB4 which is related to liver fibrosis (Supplementary Table 2).

### The dynamic of the serum lipid levels adjusted in both SVR and fibrosis groups

**Figure 2:**
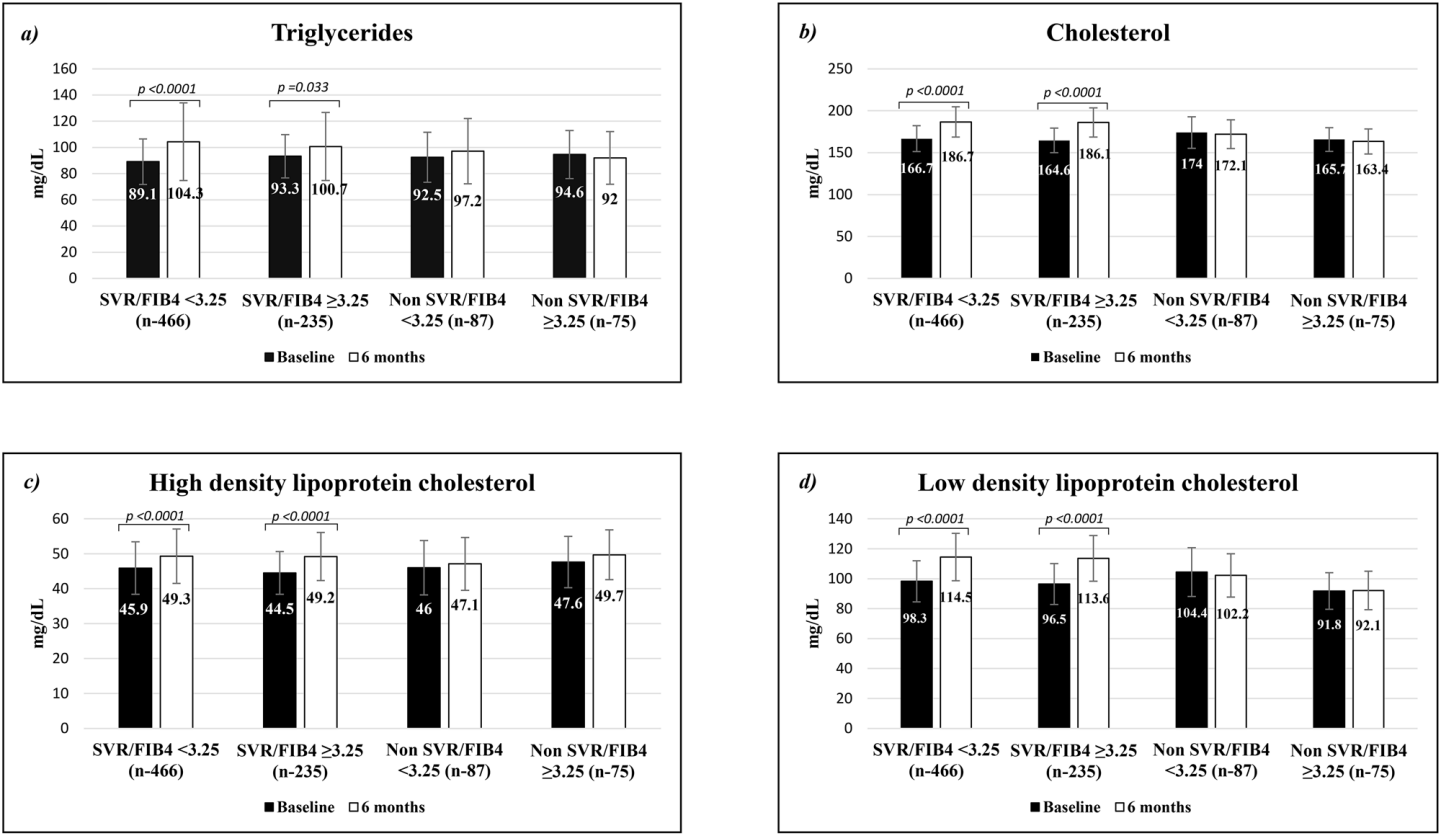
The change of serum lipid levels in baseline and after 6 months of antiviral treatment stratified by sustained virologic response and FIB4 Comparison of serum **(a)** triglycerides, **(b)** cholesterol, **(c)** high density lipoprotein cholesterol and **(d)** low density lipoprotein cholesterol levels in baseline and after 6 months of antiviral treatment.

We modified the patients in different groups by a combination of viral response and FIB4. Patients who were achieved SVR and lower FIB4 index had significant increase of lipid profiles after the treatment. These results showed SVR related increase of serum lipid profiles (Figure 2). If we look at the difference of before and after treatment in SVR patients, only serum TG level was increased more in low FIB4 group (15.1±53.3) compared to high FIB4 (6.7±47.9; p=0.041) groups (Supplementary Table 3). We performed multivariate logistic regression analysis to check associated factors to increase of serum TG levels in SVR patients. Lower FIB4 index significantly associated with more increase of serum TG levels after treatment (OR – 0.924, 95% CI – 0.859-0.993, p=0.032) adjusted with male gender and BMI (Data not shown).

However high blood pressure (hypertension) and BMI are crucial factors related to the serum lipid levels, we also analyzed before and after treatment lipid levels in hypertension or BMI stratified groups. The impact of SVR in lipid levels were still better than hypertension or BMI (Supplementary Tables 4, 5, 6 and 7).

## DISCUSSION

In this study, the association between serum lipid profiles and PegINF/RBV treatment response was described firstly in Taiwanese patients who achieved SVR by PegINF/RBV treatment. We found that a successful HCV eradication (SVR) has an impact on the lipid levels after PegINF/RBV treatment. Serum lipids were increased in patients who achieved SVR with or without fibrosis compared to non SVR patients. We also have reported that serum TG and TC were significantly lower in group of anti-HCV positive with HCV RNA than anti-HCV positive with HCV RNA negative which was performed in large scale community based study of 11,239 residents in Taiwan [13] and the serum lipids were lower in HCV infected patients compared with non HCV control group [1]. Also several clinical studies were demonstrated that lower serum cholesterol associated with HCV infection [14, 15]. Serum TG level was lower before the initiation of PegIFN/RBV treatment and increased after the treatment in our Asian cohort. Although this change was reported in HCV and human immunodeficiency virus co-infected patients [16]. The low density lipoprotein receptor is one the receptors to bind HCV and might induce entering HCV into the hepatocytes. LDLC in the serum may regulate the binding of HCV to hepatocyte receptors [17]. All these studies suggested the baseline lipid levels are lower in HCV infected individuals and HCV influence the lipid metabolism.

The decrease of lipid profiles may cause the following mechanisms. TG and TC are the crucial particles which involve HCV replication has been studied [18]. HCV nonstructural proteins were surrounded lipid droplets, which contains TG and TC, in 60-90% of HCV infected cell lines that increased replication [19]. Also it has been studied that HCV core protein have some associations in lipid metabolism which binds to apolipoprotein AII and inhibits microsomal triglyceride transfer protein to increase lipid droplets inside the cell [20], activate the Sterol-regulatory element-binding protein (SREBP) [21] and SREBP associated with increase of cholesterol load and upregulate de novo cholesterol synthesis [22]. However in clinical study apolipoprotein B had a correlation to HCV viral load, which suggested the beta-lipoproteins have an interaction with HCV [23]. Whether HCV infected cells might decrease serum lipid levels because HCV itself needs lipids in HCV infection but still need further investigation to confirm that.

It's already been described that the role of HCV core protein to decrease serum lipid levels but in the other hand IFN may alter lipid metabolism [24]. The dynamic of lipid change before and after PegIFN/RBV treatment was different in SVR and non SVR groups. It is may supported that the IFN may inhibit hepatic TG lipase which is enzyme to degrade serum TG level also IFN can stimulate TG synthesis in the hepatocyte during treatment [24]. Naeem M et al reported that baseline serum TG level was low but it is increased during INF based treatment and returned to normal after the treatment. They explained it is due to cessation of IFN treatment [25]. In the present study the serum TG level was increased significantly in SVR group. It is more related to the successful eradication of HCV is might normalizing the cellular function of hepatocyte to improve hepatic lipid production.

Our results have indicated that the HCV RNA clearance may indeed increase the lipid levels which reflected the strong association and interaction between HCV itself and the lipid levels with or without fibrosis. We also observed that non SVR patients associated with decreased TC and TG after antiviral treatment because of the continuing HCV infection.

Some studies revealed that patients with baseline high LDLC level had greater SVR rate and LDLC level decreased in significantly in non SVR group [26]. However baseline LDLC was significantly higher in low FIB4 group compared with high FIB4 group in this study, but we did not found baseline difference associated with SVR. Gopal K et al [27] also reported that baseline high TC and LDLC associated with higher odds ratio to achieve SVR. But our study did not found that baseline lipid levels were related with the SVR. Tada et al found that serum TG, TC, and LDLC levels were increased in HCV genotype 1 patients after interferon based treatment [28]. In our study HCV genotype difference in serum lipid was not changed.

We also demonstrate that advanced liver fibrosis and non SVR patients had decreased TG and TC levels but no change in HDLC and LDLC after PegIFN/RBV treatment. The change of pretreatment and post treatment TG level of patients who achieved SVR and mild fibrosis group was increased significantly, but patients with non SVR and advanced fibrosis group was decreased. The number of cells maybe too low to influence serum lipid change in advanced fibrosis patients after treatment. It is also constant if the patients did not cured HCV infection. The accumulation of lipids in hepatocyte (steatosis) is one of the common manifestations of CHC patients and the half of the HCV infected patients have steatosis. Steatosis was correlated with the progression of liver fibrosis in HCV infection [29]. Also intrahepatic HCV core protein and development of steatosis have close relationship in liver biopsy samples [30]. However the patients with moderate, severe hepatic steatosis and HCV genotype 3 were achieved complete disappearance of steatosis after IFN based treatment [31] but patients with HCV genotype 3 were extremely low in our study. The eradication of HCV influence to normalize the lipid metabolism and liver functions. This may result the stabilization of liver disease progression.

Our study has some limitations. First, we cannot exclude an influence of interferon therapy on the serum lipid levels in our treated patients. Second, our study did not give the chance to assess the condition of diet, physical activity, waist circumference and family history which are crucial for the lipid metabolism. Further studies for the evaluation of these factors are mandatory.

In conclusion, we found that increased lipid profiles including TG, TC, HDLC, LDLC levels after PegIFN/RBV treatment for CHC patients achieving SVR. Our study supports that clearance of the HCV RNA is the main determinant of the increase of lipids after PegIFN/RBV treatment. Further investigation is needed to elucidate the mechanism of lipids in fibrosis development. By the way, the impact of the increase of the lipid levels in patients with SVR needs to be studied in the future.

## PATIENTS AND METHODS

### Patients

In this retrospective study we consequently enrolled 863 patients who completed the treatment of PegIFN/RBV combination therapy in Hepatobilliary Division of Kaohsiung Medical University Hospital from 2004 to 2015. The inclusion criteria were following: a) adult patients over 18 years, b) all detectable serum HCV RNA, c) HCV treatment naïve patients. Exclusion criteria were: a) co-infected patients with hepatitis B, D or human immunodeficiency virus, b) patients who did not completed PegIFN/RBV therapy c) patients who had the information about using lipid lowering drug d) patients with extremely high TG levels (more than 2 fold increase of upper normal limit), e) past or current history of alcohol use (ethanol consumption of ≥20g per day for men and ≥10g for women), f) presence of decompensated liver cirrhosis or hepatocellular carcinoma.

### Treatment regimen

All patients in this study received either PegIFN α-2a (Pegasys, Hoffmann-La Roche, Basal, Switzerland) 180 μg/week or Peg-IFN α-2b (PEG-Intron, Schering-Plough Inc., Kenilworth, NJ, USA) 1.5 μg/kg/week plus weight-based RBV (1000 mg/d for <75 kg patients and 1200 mg/d for ≥75 kg respectively). All the patients were treated by the duration of 24-48 weeks according to a response-guided therapy (RGT) based on the HCV genotype, viral loads and viral response.

### Laboratory tests

Serum HCV RNA was detected using standardized automated qualitative reverse transcription-polymerase chain reaction assay (COBAS AMPLICOR Hepatitis C virus test, version 2.0; Roche, Branchburg, NJ, USA (Detection limit – 50IU/mL)).

Patients were checked with serum TG, total cholesterol (TC), high density lipoprotein cholesterol (HDLC) and LDLC levels pretreatment, end of the treatment and after 6 months of the cessation of the anti-HCV treatment. All lipid profiles measured by multichannel autoanalyser (Hitachi, Inc, Tokyo, Japan) using standardized enzymatic procedures and evaluated TG (mg/dL), TC (mg/dL), HDLC (mg/dL) and LDLC (mg/dL). Fibrosis stage has performed by liver biopsy and evaluated by METAVIR score.

Sustained virological response (SVR) was defined as negative HCV RNA at 6-month after cessation of treatment. The four indexes for fibrosis (FIB4) was calculated following formula and used 3.25 as a cut-off point for predict fibrosis 3 and 4 [32]. FIB4 = (age [years] x GOT [U/L]) / (Platelet [10^9^/l] x √GPT [U/L]). Each participant obtained written informed consent.

### Statistical analysis

Mean value and standard deviation were calculated in continuous variables. The continuous variables compared by Student's T test (the independent T and paired T test), Chi-square (X^2^) or Fisher's exact test were used for categorical variables. Pretreatment and post treatment groups were analyzed by univariate analysis and performed to evaluate relationship between lipid levels and FIB4 index. A P-value of less than 0.05 was considered to be statistically significant. All calculations were performed in the IBM SPSS Statistics for Windows, Version 20.0. Armonk, NY: IBM Corp.

## SUPPLEMENTARY MATERIALS FIGURES AND TABLES


