# Supplementary material for: The effect of antiviral therapy on serum lipid profiles in chronic hepatitis C
Source: Oncotarget. 2018 Apr 20;9(30):21313–21. doi: 10.18632/oncotarget.25092 (PMC5940400; doi:10.18632/oncotarget.25092)
Supplement: Supplementary file 1 [file oncotarget-09-21313-s001.pdf]

The effect of antiviral therapy on serum lipid profiles in chronic hepatitis C

SUPPLEMENTARY MATERIALS

Supplementary Table 1: Increase of lipid levels after the treatment in SVR and Non SVR groups compared to baseline lipid levels and the differences

Characteristics		Baseline	6 months	*P* value	Δ	*P* value
**Triglycerides**	n-863	91.1±34.9	101.4±55.1	**0.0001**		**0.012**
	SVR(n-701)	90.5±34.3*	102.9±57.0	**0.0001**	12.3±51.7	
	Non SVR (n-162)	93.5±37.3*	94.5±45.6	0.690	1.3±43.2	
**Total cholesterol**	n-863	166.8±31.1	183.1±35.9	**0.0001**		**0.0001**
	SVR(n-701)	166.0±30.3#	186.5±35.7	**0.0001**	20.4±30.5	
	Non SVR (n-162)	170.2±34.0#	168.1±32.5	0.352	-2.0±28.0	
**HDLC**	n-863	45.7±14.4	49.1±14.9	**0.0001**		**0.021**
	SVR(n-701)	45.4±14.2$	49.3±15.0	**0.0001**	3.8±11.5	
	Non SVR (n-162)	46.7±15.1$	48.3±14.7	0.066	1.5±10.6	
**LDLC**	n-863	97.9±27.9	111.1±31.3	**0.0001**		**0.0001**
	SVR(n-701)	97.7±27.4^	114.2±31.3	**0.0001**	16.4±27.9	
	Non SVR (n-162)	98.6±29.6^	97.5±27.5	0.596	-1.0±24.5	

HDLC – High density lipoprotein cholesterol, LDLC – low density lipoprotein cholesterol, Δ - the difference of lipid levels (shown the mean and SD), * - not significant, # - not significant, $ - not significant, ^ - not significant.

Supplementary Table 2: Increase of lipid levels after the treatment in low and high FIB4 groups compared to baseline lipid levels and the differences

**Characteristics**		**Baseline**	**6 months**	***P* value**	**Δ**	***P* value**
**Triglycerides**	n-863	91.1±34.9	101.4±55.1	**0.0001**		
	FIB4 <3.25 (n-553)	89.7±35.4*	103.2±57.9	**0.0001**	13.5±51.9	**0.011**
	FIB4 ≥3.25 (n-310)	93.6±33.9*	98.1±49.6	0.095	4.4±47.1	
**Total cholesterol**	n-863	166.8±31.1	183.1±35.9	**0.0001**		
	FIB4 <3.25 (n-553)	167.9±32.1#	184.4±36.3	**0.0001**	16.5±30.9	0.722
	FIB4 ≥3.25 (n-310)	164.9±29.0#	180.6±34.9	**0.0001**	15.7±32.0	
**HDLC**	n-863	45.7±14.4	49.1±14.9	**0.0001**		
	FIB4 <3.25 (n-553)	45.9±15.1$	49.0±15.5	**0.0001**	3.0±11.7	0.211
	FIB4 ≥3.25 (n-310)	45.3±12.9$	49.3±13.9	**0.0001**	4.0±10.7	
**LDLC**	n-863	97.9±27.9	111.1±31.3	**0.0001**		
	FIB4 <3.25 (n-553)	99.3±28.4^	112.6±31.6	**0.0001**	13.2±28.6	0.913
	FIB4 ≥3.25 (n-310)	95.4±26.6^	108.4±30.7	**0.0001**	13.0±27.3	

HDLC – High density lipoprotein cholesterol, LDLC – low density lipoprotein cholesterol, Δ - the difference of lipid levels (shown the mean and SD), * - not significant, # - not significant, $ - not significant, ^- *p*=0.047.

Supplementary Table 3: Mean of baseline and 6 months after treatment lipid levels in advanced and mild fibrosis for SVR and Non SVR patients

Characteristics	Treatment response and FIB4	Baseline	6 months	*P* value	Δ SVR	*P* value	Δ non SVR	*P* value
**Triglycerides**	SVR/FIB4 <3.25 (n-466)	89.1±34.8	104.3±59.3	**0.0001**	15.1±53.3	**0.041**		
	SVR/FIB4 ≥3.25 (n-235)	93.3±33.1	100.7±52.1	**0.033**	6.7±47.9			
	Non SVR/FIB4 <3.25 (n-87)	92.5±38.1	97.2±49.8	0.304			4.6±42.3	0.292
	Non SVR/FIB4 ≥3.25 (n-75)	94.6±36.6	92.0±40.2	0.624			-2.5±44.1	
**Total cholesterol**	SVR/FIB4 <3.25 (n-466)	166.7±30.9	186.7±36.3	**0.0001**	19.9±30.4	0.533		
	SVR/FIB4 ≥3.25 (n-235)	164.6±29.3	186.1±34.6	**0.0001**	21.5±30.7			
	Non SVR/FIB4 <3.25 (n-87)	174.0±37.8	172.1±34.2	0.524			-1.8±27.2	0.927
	Non SVR/FIB4 ≥3.25 (n-75)	165.7±28.4	163.4±30.0	0.501			-2.2±29.1	
**HDLC**	SVR/FIB4 <3.25 (n-466)	45.9±15.0	49.3±15.6	**0.0001**	3.4±11.9	0.167		
	SVR/FIB4 ≥3.25 (n-235)	44.5±12.2	49.2±13.8	**0.0001**	4.6±10.5			
	Non SVR/FIB4 <3.25 (n-87)	46.0±15.6	47.1±15.1	0.329			1.0±10.2	0.549
	Non SVR/FIB4 ≥3.25 (n-75)	47.6±14.7	49.7±14.3	0.108			2.0±11.1	
**LDLC**	SVR/FIB4 <3.25 (n-466)	98.3±27.5	114.5±31.7	**0.0001**	16.1±28.7	0.674		
	SVR/FIB4 ≥3.25 (n-235)	96.5±27.3	113.6±30.5	**0.0001**	17.1±26.4			
	Non SVR/FIB4 <3.25 (n-87)	104.4±32.5	102.2±28.9	0.366			-2.2±22.7	0.508
	Non SVR/FIB4 ≥3.25 (n-75)	91.8±24.4	92.1±25.7	0.908			0.3±26.6	

HDLC – High density lipoprotein cholesterol, LDLC – low density lipoprotein cholesterol, Δ - the difference of lipid levels (shown the mean and SD).

Supplementary Table 4: Increase of lipid levels after the treatment in high and normal blood pressure groups compared to baseline lipid levels and the differences

**Characteristics**		**Baseline**	**6 months**	***P* value**	**Δ**	***P* value**
**Triglycerides**	n-816	91.3±34.8	101.5±55.4	**0.0001**		0.353
	Hypertension (n-194)	97.6±36.0*	110.7±55.2	**0.0001**	13.0±49.8	
	Normal (n-622)	89.4±34.2*	98.7±55.1	**0.0001**	9.2±50.0	
**Total cholesterol**	n-816	166.7±31.2	183.0±36.1	**0.0001**		**0.031**
	Hypertension (n-194)	169.4±30.8#	181.4±35.5	**0.0001**	12.0±33.5	
	Normal (n-622)	165.8±31.3#	183.4±36.3	**0.0001**	17.6±30.7	
**HDLC**	n-816	45.5±14.4	49.2±15.1	**0.0001**		**0.040**
	Hypertension (n-194)	45.4±14.4$	47.5±13.8	**0.007**	2.1±11.2	
	Normal (n-622)	45.5±14.4$	49.7±15.5	**0.0001**	4.1±11.5	
**LDLC**	n-816	97.7±28.0	110.9±31.4	**0.0001**		0.195
	Hypertension (n-194)	99.2±29.2^	110.0±31.5	**0.0001**	10.8±28.8	
	Normal (n-622)	97.3±27.6^	111.1±31.4	**0.0001**	13.8±28.1	

HDLC – High density lipoprotein cholesterol, LDLC – low density lipoprotein cholesterol, Δ - the difference of lipid levels (shown the mean and SD), * - p=0.004, # - not significant, $ - not significant, ^- not significant.

Supplementary Table 5: Mean of baseline and 6 months after treatment lipid levels in high and normal blood pressure for SVR and Non SVR patients

Characteristics	Treatment response and blood pressure	Baseline	6 months	*P* value	Δ SVR	*P* value	Δ non SVR	*P* value
**Triglycerides**	SVR/Normal (n-515)	89.2±34.0	100.4±57.8	**0.0001**	11.1±51.4	0.255		
	SVR/Hypertension (n-145)	95.8±34.1	112.5±56.0	**0.0001**	16.7±51.8			
	Non SVR/ Normal (n-107)	90.2±35.1	90.3±39.1	0.985			0.1±41.5	0.749
	Non SVR/ Hypertension (n-49)	103.0±40.8	105.4±53.2	0.693			2.3±42.0	
**Total cholesterol**	SVR/Normal (n-515)	165.9±31.2	187.3±35.8	**0.0001**	21.3±29.8	0.260		
	SVR/Hypertension (n-145)	165.5±27.7	183.7±36.6	**0.0001**	18.1±33.5			
	Non SVR/ Normal (n-107)	165.5±32.3	165.1±33.0	0.893			-0.3±28.6	0.251
	Non SVR/ Hypertension (n-49)	180.7±36.5	174.7±31.2	0.125			-5.9±26.6	
**HDLC**	SVR/Normal (n-515)	45.5±14.4	50.1±15.5	**0.0001**	4.5±11.5	0.093		
	SVR/Hypertension (n-145)	44.3±13.5	47.0±13.6	**0.006**	2.6±11.6			
	Non SVR/ Normal (n-107)	45.6±14.6	47.7±15.3	**0.042**			2.1±11.0	0.415
	Non SVR/ Hypertension (n-49)	48.6±16.4	49.3±14.3	0.618			0.6±9.6	
**LDLC**	SVR/Normal (n-515)	97.4±27.2	114.1±31.4	**0.0001**	16.7±27.7	0.593		
	SVR/Hypertension (n-145)	98.2±28.6	113.5±31.8	**0.0001**	15.3±29.3			
	Non SVR/ Normal (n-107)	96.8±29.2	96.6±27.6	0.922			-0.2±25.6	0.583
	Non SVR/ Hypertension (n-49)	102.3±31.1	99.7±28.6	0.433			-2.6±23.0	

HDLC – High density lipoprotein cholesterol, LDLC – low density lipoprotein cholesterol, BMI – body mass index, SVR – sustained virological response.

Supplementary Table 6: Increase of lipid levels after the treatment in high and normal BMI groups compared to baseline lipid levels and the differences

**Characteristics**		**Baseline**	**6 months**	***P* value**	**Δ**	***P* value**
**Triglycerides**	n-863	91.1±34.9	101.4±55.1	**0.0001**		0.843
	High BMI (n-391)	99.0±35.5*	109.7±53.6	**0.0001**	10.6±51.6	
	Normal BMI (n-472)	84.5±33.0*	94.5±55.4	**0.0001**	9.9±49.4	
**Total cholesterol**	n-863	166.8±31.1	183.1±35.9	**0.0001**		**0.003**
	High BMI (n-391)	169.0±29.5#	181.8±35.8	**0.0001**	12.7±30.2	
	Normal BMI (n-472)	165.0±32.2#	184.1±35.9	**0.0001**	19.1±31.9	
**HDLC**	n-863	45.7±14.4	49.1±14.9	**0.0001**		0.873
	High BMI (n-391)	43.4±13.4$	46.7±14.6	**0.0001**	3.3±11.0	
	Normal BMI (n-472)	47.6±14.9$	51.1±14.9	**0.0001**	3.4±11.7	
**LDLC**	n-863	97.9±27.9	111.1±31.3	**0.0001**		**0.042**
	High BMI (n-391)	100.9±27.6^	111.9±31.8	**0.0001**	11.0±28.9	
	Normal BMI (n-472)	95.4±27.8^	110.4±30.9	**0.0001**	14.9±27.4	

HDLC – High density lipoprotein cholesterol, LDLC – low density lipoprotein cholesterol, BMI – body mass index, Δ - the difference of lipid levels (shown the mean and SD), * - $p<0.0001$, # - not significant, $ - $p<0.0001$, ^- $p=0.004$.

Supplementary Table 7: Mean of baseline and 6 months after treatment lipid levels in high and normal BMI for SVR and Non SVR patients

Characteristics	Treatment response and blood pressure	Baseline	6 months	*P* value	Δ SVR	*P* value	Δ non SVR	*P* value
**Triglycerides**	SVR/Normal BMI (n-403)	83.9±32.0	97.0±58.5	**0.0001**	13.0±51.2	0.673		
	SVR/High BMI (n-298)	99.4±35.3	110.8±53.9	**0.0001**	11.3±52.5			
	Non SVR/Normal BMI (n-69)	87.7±38.3	79.7±26.9	**0.044**			-8.0±32.4	**0.011**
	Non SVR/High BMI (n-93)	97.7±36.1	106.0±52.9	0.103			8.3±48.7	
**Total cholesterol**	SVR/Normal BMI (n-403)	164.2±31.0	186.9±35.9	**0.0001**	22.7±30.6	**0.020**		
	SVR/High BMI (n-298)	168.5±29.3	185.9±35.6	**0.0001**	17.3±30.1			
	Non SVR/Normal BMI (n-69)	169.6±38.3	167.4±31.3	0.556			-2.2±31.4	0.942
	Non SVR/High BMI (n-93)	170.5±30.6	168.6±33.6	0.468			-1.9±25.3	
**HDLC**	SVR/Normal BMI (n-403)	47.3±14.7	51.4±15.1	**0.0001**	4.0±11.7	0.590		
	SVR/High BMI (n-298)	42.8±12.9	46.4±14.4	**0.0001**	3.5±11.3			
	Non SVR/Normal BMI (n-69)	49.0±15.6	49.1±13.9	0.940			0.1±11.1	0.137
	Non SVR/High BMI (n-93)	45.0±14.6	47.6±15.4	**0.015**			2.6±10.1	
**LDLC**	SVR/Normal BMI (n-403)	94.9±27.2	112.5±31.2	**0.0001**	17.6±26.8	0.213		
	SVR/High BMI (n-298)	101.5±27.4	116.5±31.4	**0.0001**	14.9±29.3			
	Non SVR/Normal BMI (n-69)	98.2±31.5	97.7±25.9	0.881			-0.4±25.7	0.804
	Non SVR/High BMI (n-93)	98.8±28.3	97.4±28.8	0.560			-1.4±23.8	

HDLC – High density lipoprotein cholesterol, LDLC – low density lipoprotein cholesterol, BMI – body mass index, SVR – sustained virological response.
